# Effects of the use of clinical simulators on the development of practical skills in higher education

**DOI:** 10.3389/fmed.2026.1785729

**Published:** 2026-04-08

**Authors:** Edwin Julio Cóndor-Salvatierra, Ricardo Ángel Yuli-Posadas, Melva Iparraguirre-Meza, Mildred Hilda Condor-Privat, Beatriz Lilian Galdós-Vadillo, Ivonne Roció Poma-Mansilla, Lolita Arévalo-Fasanando, Edgar Robert Tapia-Manrique, Antonio José Obregón-La Rosa, José Miguel Rutti-Marin

**Affiliations:** 1 Facultad de Ciencias de la Educación, Universidad Nacional de Huancavelica, Huancavelica, Peru; 2 Facultad de Farmacia y Bioquímica, Universidad Nacional Mayor de San Marcos, Lima, Peru; 3 Facultad de Ciencias de la Salud, Universidad Peruana Los Andes, Huancayo, Peru; 4 Facultad de Ciencias de la Salud, Universidad Nacional de San Martin, San Martin, Peru; 5 Facultad de Ciencias de la Salud, Universidad Tecnológica del Perú, Lima, Peru; 6 Facultad de Ingeniería, Universidad Nacional Intercultural de la Selva Central Juan Santos Atahualpa, La Merced, Peru

**Keywords:** clinical simulation, medical education, obstetrics, professional competencies, university training

## Abstract

**Introduction:**

Clinical simulation has been consolidated as an innovative strategy to strengthen practical teaching in higher education in health sciences, as it enables the integration of theoretical knowledge with the development of clinical competencies in safe and controlled environments. The objective of the study was to analyze the effects of the use of clinical simulators on the development of practical skills among students of the Professional School of Obstetrics at Universidad Peruana Los Andes.

**Methodology:**

A quantitative, applied, descriptive–correlational design was employed. The sample consisted of 226 students selected through stratified probabilistic sampling, considering academic cycle and gender as strata to ensure representativeness of the student population. Data were collected using a 30-item structured questionnaire distributed across five theoretical dimensions, measured through a five-point Likert scale. The reliability of the instrument was assessed using Cronbach’s Alpha, obtaining an overall coefficient of *α* = 0.978, which indicates excellent internal consistency. Data analysis included descriptive and inferential statistics using IBM SPSS version 26, and statistical significance was established at *p* < 0.05.

**Results:**

The results showed that the dimensions “teamwork and communication” (*β* = 0.296; *p* = 0.005) and “safety and confidence in clinical performance” (*β* = 0.613; *p* < 0.001) had statistically significant relationships with the overall perception of formative impact, whereas the dimensions “access and use” and “technical competencies” did not show significant effects. These findings confirm that clinical simulation primarily influences the strengthening of socio-emotional and collaborative competencies in professional training.

**Conclusion:**

Clinical simulation constitutes an effective pedagogical strategy to enhance students’ self-confidence, professional safety, and the practical integration of knowledge in health education. Its incorporation into university curricula is recommended, along with further research assessing its long-term impact on clinical performance.

## 1 Introduction

The integration of clinical simulators into university education has emerged as a pedagogical strategy that transforms traditional teaching into controlled, safe, and repeatable practice environments (1, 2). The first developments in this field date back to the 1960s with the creation of Sim One, a pioneer in the physiological reproduction of human functions, allowing students to practice anesthetic procedures without direct risk to patients (3). Over time, technological advances have facilitated the incorporation of high-fidelity simulators that realistically replicate critical situations such as obstetric complications, cardiovascular emergencies, and surgical procedures. This has enabled future professionals to acquire skills in scenarios that are difficult to experience in real clinical practice. Research has shown that these methodologies not only strengthen the acquisition of technical skills but also promote students’ confidence, clinical judgment, and teamwork abilities (4, 5).

Clinical simulation has become a fundamental pedagogical strategy in the education of health professionals because it enables the integration of theoretical knowledge, technical skills, and socio-emotional competencies within controlled learning environments that replicate real clinical situations. Several studies indicate that simulation-based education facilitates the development of cognitive, psychomotor, and affective skills by providing experiential learning scenarios that promote clinical decision-making and critical reasoning in safe environments for students (6). From this perspective, simulation should not be considered merely as a complementary instructional tool but rather as a pedagogical environment that enables the articulation of theoretical knowledge with professional practice. In this sense, its integration into health training programs contributes to strengthening the progressive development of professional competencies, particularly those related to clinical judgment, communication, and collaborative work in complex healthcare settings. Furthermore, from a constructivist pedagogical perspective, simulation environments are understood as intermediate spaces between the classroom and real clinical practice, facilitating students’ gradual transition to professional practice through contextualized learning experiences (2). Consequently, clinical simulation emerges as a key educational resource for reducing the traditional gap between theory and practice in the training of health professionals.

In the field of higher education in health sciences, multiple studies have demonstrated that the use of clinical simulators contributes to reducing medical errors and improving emergency response (7, 8). In obstetrics, for instance, their implementation has shown a positive impact in decreasing neonatal injuries associated with shoulder dystocia and in refining maneuvers for postpartum hemorrhage management (9, 10). Likewise, recent research indicates that simulators foster the transition between theoretical knowledge and practical application, optimizing the preparation of nursing and obstetrics students before facing real clinical scenarios (11). Within this context, the use of clinical simulators is consolidated as an indispensable resource for the development of practical skills in higher education, ensuring patient safety and the training of competent professionals capable of meeting the challenges of contemporary clinical practice.

At the international level, several studies have identified significant gaps in the clinical preparedness of future health professionals, particularly in managing obstetric emergencies. In low- and middle-income countries (LMICs), which account for approximately 99% of maternal deaths, the implementation of clinical simulation faces limitations due to lack of funding, infrastructure, and trained personnel (12). Although simulation has proven effective in reducing critical complications such as shoulder dystocia, where neonatal injury rates decreased from 9.3 to 2.3% following regular training challenges persist in establishing sustainable programs (11). Additionally, it has been documented that 30% of maternal deaths in resource-limited settings are due to postpartum hemorrhage, many of which could be prevented through simulation-based training (13). These findings highlight a gap between the demonstrated potential of clinical simulation and its effective implementation in global medical education.

In the Peruvian context, the problem is intensified by the need for newly graduated physicians to face obstetric emergencies in rural areas under the SERUMS program, often without multidisciplinary support teams. A study conducted in Lima with 463 newly graduated physicians revealed that only 33.3% considered themselves competent to manage obstetric emergencies, a percentage that dropped to 35.0% for preeclampsia management and 54.1% for postpartum hemorrhage (14). However, low-cost interventions such as supervised daily practices, weekly simulations, and brief monthly training sessions increased the pass rates in objective clinical evaluations from 83 to 95% over 6 months, demonstrating the effectiveness of simulation in counteracting skills attrition in resource-limited contexts (15). This finding underscores the urgent need to integrate simulation as a permanent pedagogical tool, particularly in rural regions with limited health infrastructure. These gaps reflect not only deficiencies in technical training but also biases and discriminatory practices that limit equity in healthcare. Furthermore, recent studies show that, although the implementation of simulation training courses for instructors in Peruvian universities has achieved satisfaction levels above 66% among participants, difficulties persist in transferring what is learned into practice due to academic disorganization and insecurities in applying debriefing methodologies (16).

Despite the growing recognition of the value of clinical simulation in health education, the scientific literature indicates that there are still limitations in the systematic evaluation of its impact on the development of practical competencies. Recent studies suggest that a large proportion of research on simulation has primarily focused on analyzing students’ perceptions, satisfaction levels, or confidence regarding simulation-based learning experiences, whereas empirical investigations examining the relationship between the use of clinical simulators and the effective development of measurable professional skills remain limited (6). This situation highlights the need to strengthen methodological approaches capable of objectively analyzing learning outcomes derived from the use of simulation in higher health education. In addition, recent research on educational technologies in medical training suggests that the integration of technological environments and immersive simulations can enhance students’ clinical confidence and overall learning experience (17). However, these findings also underscore the necessity for further research examining how these tools influence the development of practical competencies in specific educational contexts. Therefore, a research gap exists regarding the empirical evaluation of the impact of clinical simulators on the development of practical skills in obstetrics students, which justifies the need for studies aimed at analyzing this relationship within specific university contexts.

The existing literature on the use of clinical simulators in university education shows significant advances in high-income countries. However, in the Peruvian context, notable gaps remain regarding the lack of systematic studies evaluating their impact on the acquisition and retention of practical skills in real healthcare scenarios. Available national research has mainly focused on short-term training or participant satisfaction, without delving into the longitudinal effectiveness of simulation in reducing performance gaps or its potential to mitigate ethnic and structural inequalities in healthcare delivery. In this sense, Universidad Peruana Los Andes, as a leading institution in health professional training in Peru, represents a strategic space for developing such studies not only because of its historical role in higher education but also due to its capacity to generate scientific evidence that contributes to strengthening public policies and pedagogical practices ensuring high-quality and socially relevant clinical education.

Within this framework, the research question is formulated as follows: What is the effect of the use of clinical simulators on the development of practical skills in students of the Professional School of Obstetrics at Universidad Peruana Los Andes? Accordingly, the following hypotheses are proposed:

*H1*. Access to and use of clinical simulators are significantly and positively related to the overall perception and formative impact among obstetrics students.

*H2*. The acquisition of technical competencies derived from the use of clinical simulators is positively associated with the overall perception and formative impact.

*H3*. Teamwork and communication developed during simulations positively influence the overall perception and formative impact.

*H4*. The safety and confidence gained in clinical performance through simulation are directly and significantly related to the overall perception and formative impact.

Accordingly, the objective of the study was to analyze the effects of the use of clinical simulators on the development of practical skills among students of the Professional School of Obstetrics at Universidad Peruana Los Andes, in order to provide scientific evidence to guide curricular improvements and promote educational quality in the field of health.

## 2 Methods

The study followed a quantitative approach with a non-experimental, correlational research design, aimed at analyzing the relationship between the use of clinical simulators and the development of practical skills among students in higher education. This design allowed the observation of the variables in their natural context without manipulation, enabling the identification of potential associations between the use of simulation-based learning resources and the development of practical competencies in clinical training. The study population consisted of students enrolled in the Obstetrics program at Universidad Peruana Los Andes during the academic period of the study, who met the inclusion criteria established for the research. To ensure representativeness, a simple random sampling technique was used, allowing all members of the population to have an equal probability of being selected.

The final sample consisted of 226 students, selected from the study population. It should be noted that only participants who completed the research instrument in its entirety were included in the final analysis. Questionnaires with incomplete responses or inconsistent information were excluded to ensure the reliability and validity of the data used in the statistical analysis. The sample size was calculated using the statistical formula for finite populations, considering a confidence level of 95%, a margin of error of 5%, and an expected proportion of 0.5, which is commonly used when population variability is unknown. Based on these parameters, a minimum sample size of 226 participants was determined. The selection of participants was carried out through a simple random sampling procedure, ensuring that each member of the population had an equal probability of being selected, thereby reducing potential selection bias and improving the representativeness of the sample.

Table 1 presents the sociodemographic and academic characteristics of the study participants, allowing for contextualization of the sample composition based on variables such as age, gender, academic cycle, practical experience, and prior use of clinical simulators.

**Table 1:** Sample characteristics.

Variable	Frequency	Percentage (%)
Age		
17–19 years	124	54.9
20–24 years	91	40.3
Over 24 years	11	4.9
Gender		
Female	219	96.9
Male	7	3.1
Academic cycle or semester		
First cycle	30	13.3
Second cycle	54	23.9
Third cycle	28	12.4
Fourth cycle	45	19.9
Fifth cycle	25	11.1
Sixth cycle	32	14.2
Seventh cycle	8	3.5
Ninth cycle	4	1.8
Previous clinical practice experience		
No	150	66.4
Yes	76	33.6
Prior use of clinical simulators		
No	173	76.5
Yes	53	23.5
Total	226	100.0

According to Table 1, the final sample consisted of 226 students, of whom 96.9% were female and 3.1% male, reflecting the predominant demographic composition in obstetrics-related careers. Regarding age, the largest group ranged from 17 to 19 years (54.9%), followed by 20 to 24 years (40.3%), confirming the youthful nature of the cohort and its correspondence with the initial and intermediate levels of the program. In terms of academic cycle, participation was distributed from the first to the ninth cycle, with the second (23.9%) and fourth (19.9%) being the most represented. These data demonstrate an adequate diversity in the participants’ academic trajectory, strengthening the validity of inferences regarding the educational experience with clinical simulators. Regarding practical experience, 66.4% of students reported not having previously participated in clinical practice, while 33.6% indicated prior experience. Similarly, 76.5% had not used clinical simulators before the study, compared to 23.5% who had some degree of familiarity. This distribution confirms the relevance of the analysis, as it allowed comparisons between students with different levels of practical and technological exposure.

The data collection technique consisted of a survey administered through a structured questionnaire divided into two sections. The first section addressed sociodemographic variables, while the second comprised 30 items distributed across five theoretical dimensions: (1) access and use of clinical simulators, (2) acquisition of technical competencies, (3) teamwork and communication, (4) safety and confidence in clinical performance, and (5) overall perception and formative impact. Prior to completing the questionnaire, participants were required to review an informed consent statement integrated at the beginning of the Google Form. This document detailed the purpose of the study, the voluntary nature of participation, and the guarantee of confidentiality and anonymity of the data collected. Participants could only access and answer the instrument after accepting the consent; otherwise, the form automatically ended without recording any information. This protocol ensured adherence to ethical research principles and safeguarded participants’ autonomy and data protection.

Table 2 shows the results of the reliability analysis of the instrument used for data collection, displaying the internal consistency coefficients obtained through Cronbach’s Alpha for each of the evaluated dimensions.

**Table 2:** Instrument reliability analysis.

Dimension	Cronbach’s alpha	Number of items
1. Access and use of clinical simulators	0.891	6
2. Acquisition of technical competencies	0.969	8
3. Teamwork and communication	0.966	6
4. Safety and confidence in clinical performance	0.951	5
5. Overall perception and formative impact	0.946	5
Total	0.978	30

Table 2 presents the results of the instrument reliability analysis, which yielded an overall Cronbach’s Alpha coefficient of 0.978, indicating excellent internal consistency. The dimension values ranged between 0.891 and 0.969, confirming the instrument’s stability and precision. According to psychometric standards, values above 0.80 indicate high homogeneity among items, demonstrating that the proposed dimensions robustly measured conceptually coherent constructs. Notably, the “Acquisition of technical competencies” dimension (*α* = 0.969) exhibited the highest level of consistency, reflecting that items related to clinical practice and technical proficiency were strongly correlated, reinforcing their relevance for assessing the impact of simulation on professional training.

The Kaiser–Meyer–Olkin (KMO) test of sampling adequacy (0.960) and Bartlett’s test of sphericity (*χ*^2^ = 8412.735; df = 406; *p* < 0.001) demonstrated high correlations among items, confirming the suitability of confirmatory factor analysis and strong construct validity. Factor loadings above 0.70 and model fit indices (CFI = 0.900; RMSEA = 0.098; CMIN/DF = 3.171) indicated a theoretically coherent and statistically acceptable structure. Similarly, the composite reliability (CR > 0.70) and average variance extracted (AVE > 0.50) values confirmed the convergent and discriminant validity of the dimensions, ensuring that the instrument accurately and distinctly measured the proposed constructs.

The data processing and analysis were performed using IBM SPSS version 26. Descriptive analyses (frequencies, percentages, means, and standard deviations) were first conducted to characterize the sample and describe response trends across dimensions. Subsequently, inferential analyses were developed to identify significant relationships among the main study variables.

This study was conducted in accordance with the ethical principles established in the Declaration of Helsinki (44) and international ethical guidelines for research involving human participants. The research protocol was reviewed and approved by an independent institutional ethics committee for research involving human participants, under approval number IRB-2024-0412, issued on 12 April 2024. The committee evaluated the methodological design, the data collection procedures, and the measures implemented to ensure the protection of participants’ rights, confidentiality, and data security.

For their part, the participants were previously informed about the objectives, scope, and nature of the study and voluntarily agreed to participate by providing digital informed consent integrated into the online questionnaire. Participation was entirely voluntary, and respondents were informed that they could withdraw from the study at any time without any consequences. In addition, the confidentiality and anonymity of the collected information were guaranteed, as no personally identifiable data were recorded and the information obtained was used exclusively for academic and scientific purposes.

## 3 Results and discussion

This section presents the main findings obtained after data processing and analysis, with the purpose of contrasting the stated objectives and examining the relationship between the use of clinical simulators and the development of practical skills among students. The results are interpreted in connection with previous empirical evidence, allowing the establishment of theoretical and methodological correspondences that support the validity of the findings.

**Figure 1:**
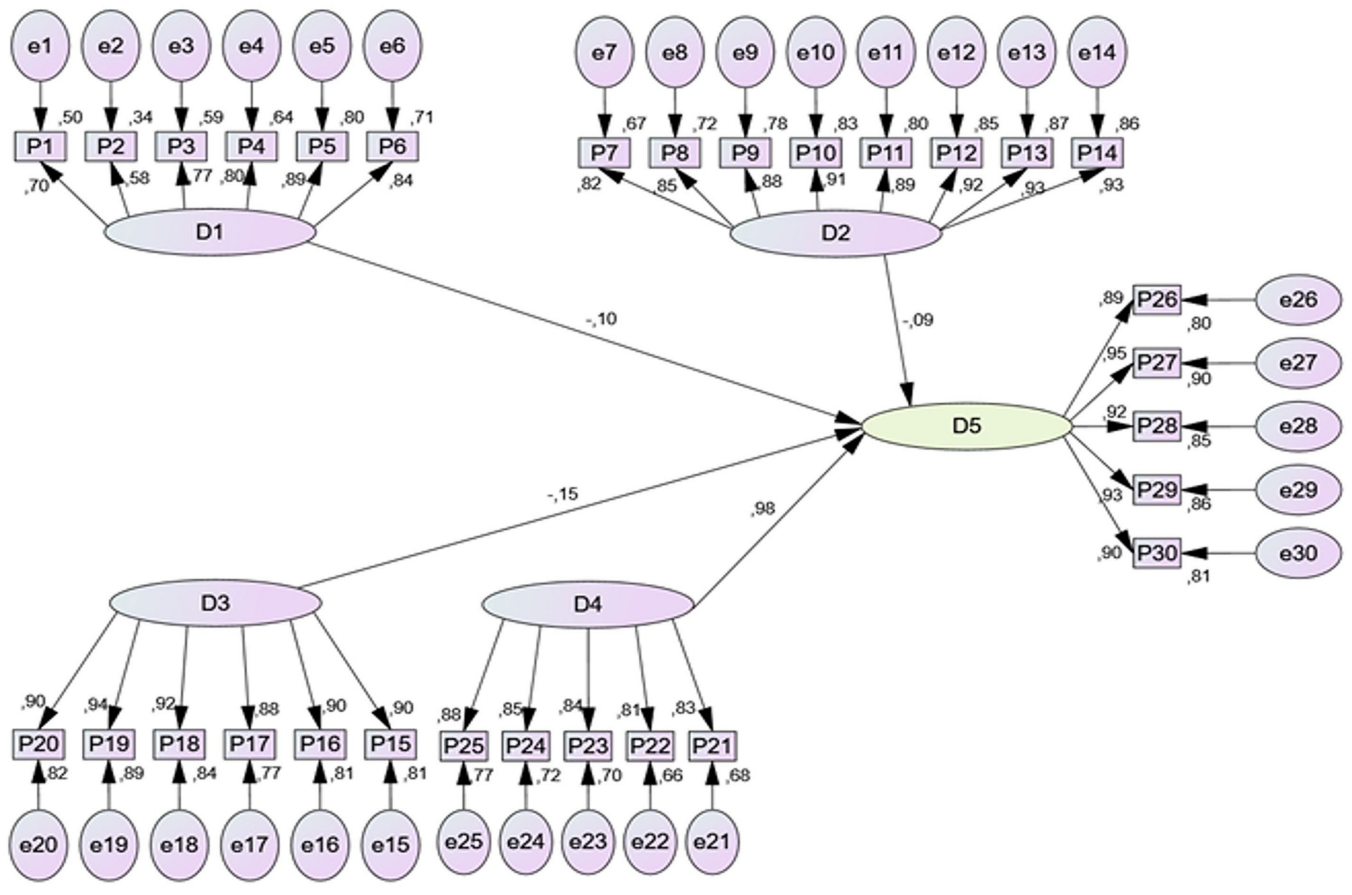
Structural equation model (PLS-SEM) used to evaluate the measurement structure of the instrument and the relationships between constructs. The figure presents the structural equation model (PLS-SEM) illustrating the relationships between the latent constructs related to the use of clinical simulators and the development of practical skills. The acronyms represent model fit indices: CFI, Comparative Fit Index; RMSEA, Root Mean Square Error of Approximation; and CMIN/DF, Chi-square divided by degrees of freedom, which evaluate the goodness of fit of the model.

Figure 1 presents the measurement structure of the research instrument used in this study, illustrating the theoretical dimensions and their corresponding indicators that assess the use of clinical simulators and the development of practical skills among students. The diagram shows the latent constructs (D1–D5) and the observed variables (P1–P30) that compose the questionnaire, as well as the relationships between each dimension and its respective indicators. This graphical representation allows visualization of how the items included in the instrument contribute to the measurement of the study variables and provides a structural overview of the constructs analyzed within the research framework.

The results illustrated in the Figure 1 confirm a direct and significant association between exposure to clinical simulation scenarios and the improvement of students’ technical and attitudinal competencies. This finding is consistent with recent studies indicating that simulation constitutes an effective pedagogical strategy to strengthen self-confidence, decision-making, and safety in clinical performance (18). Similarly, Coyne et al. (19) point out that virtual and hybrid simulation experiences facilitate the acquisition of complex clinical competencies by providing a controlled and safe environment where errors become opportunities for meaningful learning.

Furthermore, previous research in nursing and obstetrics demonstrates that simulation-based training increases students’ satisfaction and self-confidence, resulting in a more effective transfer of knowledge to the hospital setting (20, 21). These findings reaffirm that simulated environments foster reflective practice and experiential learning supported by immediate feedback, which enhances communication competence and collaborative work essential elements in comprehensive clinical care (22).

Table 3 illustrates the relationship between the level of realism in simulation scenarios and the acquisition of practical competencies among students, showing how environmental fidelity, technological resources, and active participation influence learning effectiveness. This model integrates dimensions of physical, conceptual, and emotional realism, reflecting the interaction between students’ perception and the pedagogical effectiveness of clinical simulation.

**Table 3:** Unstandardized factor loadings of the measurement model for dimensions related to the use of clinical simulators.

Questions	Connector	Dimension	Estimate	S.E.	C.R.	*p*
P1. I have had sufficient access to clinical simulators during my training	←	D1	1			
P2. Clinical simulators are available at suitable times	←	D1	0.842	0.104	8.069	^***^
P3. The university’s infrastructure facilitates the use of clinical simulators	←	D1	1.146	0.103	11.121	^***^
P4. The number of available simulators is sufficient for students	←	D1	1.109	0.098	11.351	^***^
P5. Instructors consistently promote the use of clinical simulators	←	D1	1.180	0.093	12.689	^***^
P6. Access to clinical simulators has allowed me to practice multiple times	←	D1	1.185	0.097	12.194	^***^
P8. I believe I have strengthened my ability to perform clinical procedures	←	D2	0.974	0.060	16.153	^***^
P9. Simulation has increased my accuracy in performing techniques	←	D2	1.028	0.060	17.111	^***^
P10. Simulators have allowed me to correct errors without harming real patients	←	D2	1.092	0.060	18.161	^***^
P11. Practice with simulators has increased my confidence in clinical procedures	←	D2	1.047	0.059	17.789	^***^
P12. The learning obtained is transferable to real clinical scenarios	←	D2	1.051	0.056	18.729	^***^
P13. Simulations have strengthened my clinical judgment in decision-making	←	D2	1.085	0.058	18.829	^***^
P15. Simulation has improved my ability to work in a team	←	D3	1			
P16. Communication among peers is reinforced during simulations	←	D3	0.982	0.044	22.413	^***^
P17. The dynamics of simulation promote collaboration among students	←	D3	0.978	0.047	21.005	^***^
P18. The simulator has allowed me to assume specific roles within a team	←	D3	0.983	0.042	23.599	^***^
P19. Practice in simulated settings strengthens empathy with other participants	←	D3	0.958	0.039	24.794	^***^
P20. Simulation exercises foster shared responsibility within the group	←	D3	0.965	0.044	21.698	^***^
P21. I feel more confident in facing clinical procedures after using simulators	←	D4	1			
P22. Simulation has reduced my anxiety levels during clinical emergencies	←	D4	0.950	0.047	20.290	^***^
P23. Training with simulators has prepared me to act under pressure	←	D4	0.976	0.043	22.586	^***^
P24. I consider clinical simulation essential for my professional training	←	D4	1.009	0.052	19.495	^***^
P25. Experience with simulators motivates me to continue improving my skills	←	D4	1.025	0.048	21.334	^***^
P26. The use of clinical simulators is an innovative methodology in my career	←	D5	1			
P27. Simulations represent added value to my learning process	←	D5	1.028	0.050	20.486	^***^
P28. Clinical simulation effectively complements theoretical teaching	←	D5	1.030	0.054	19.233	^***^
P29. The results obtained in the simulators reflect my academic progress	←	D5	0.980	0.055	17.671	^***^
P30. I would recommend the implementation of clinical simulators in all practical subjects	←	D5	1.044	0.062	16.914	^***^
P7. The use of clinical simulators has helped me improve my practical skills	←	D2	1			
P14. The use of clinical simulators has fostered the development of my critical thinking	←	D2	1.084	0.057	18.862	^***^

The table presents the estimates obtained from the Confirmatory Factor Analysis (CFA) for each item of the instrument, grouped by theoretical dimension. The factor loading values reflect the degree of contribution of each item to its corresponding construct, while the indicators of Standard Error (S.E.), Critical Ratio (C.R.), and significance (p) allow for the evaluation of the items’ validity within the structural model. ***p < 0.001 indicates statistically significant factor loadings at a high confidence level, confirming the robustness of the measurement model.

The results presented in Table 3 indicate that the indicators associated with the dimensions of the measurement model exhibit statistically significant factor loadings, reflecting a consistent contribution of the observed items to their respective latent constructs. The magnitude of the estimates, together with the high critical ratio values and the statistical significance levels (*p* < 0.001), confirms the robustness of the relationships between the indicators and the constructs related to the use of clinical simulators in professional training. In this regard, McGaghie et al. (23) demonstrated that simulation-based medical education supported by deliberate practice produces superior learning outcomes compared with traditional clinical instruction. This evidence supports the interpretation that simulation environments provide structured opportunities for repetitive practice, which strengthens skill acquisition and promotes the consolidation of clinical judgment through experiential learning processes in controlled and safe contexts.

Likewise, Coro-Montanet et al. (24) argue that realism, understood as the perceptual correspondence between the simulated environment and real clinical practice, constitutes a key factor for meaningful learning because it enhances immersion, self-confidence, and participant motivation. From an educational standpoint, this perspective suggests that the effectiveness of simulation does not depend solely on technological sophistication but also on the pedagogical design of scenarios that reproduce authentic professional challenges. Consequently, realistic simulations facilitate deeper cognitive engagement and allow students to internalize clinical decision-making processes, thereby strengthening their readiness to face real healthcare situations.

Similarly, Yang et al. (25) reported that simulation-based teaching methodologies significantly improve clinical judgment and critical thinking competencies compared with traditional instruction. These findings indicate that the integration of realistic scenarios, advanced simulators, and instructor feedback promotes reflective learning processes that enhance the transfer of knowledge to real clinical settings. In the same line, Cooper and Taqueti (26) highlighted that the technological evolution of high-fidelity simulators has enabled the development of interactive and reproducible training scenarios capable of preparing students for complex clinical situations. Therefore, realism in simulation should be understood as a multidimensional construct that encompasses physical fidelity, cognitive engagement, and emotional involvement, elements that collectively facilitate self-regulated learning and the consolidation of professional competencies transferable to real healthcare environments (24, 27).

Table 4 shows the structural representation of the confirmatory factor model, in which the relationships between the theoretical dimensions and the observed items of the applied instrument are visualized. This model allows for verifying construct validity by identifying standardized factor loadings and the internal coherence of the dimensions that explain the use of clinical simulators in university practical training.

**Table 4:** Confirmatory factor model of the dimensions of the use of clinical simulators.

Measure	Estimate	Threshold	Interpretation
CMIN	1,252.476	–	–
DF	395	–	–
CMIN/DF	3.171	Between 1 and 3	Acceptable
CFI	0.900	>0.95	Acceptable
RMSEA	0.098	<0.06	Poor
PClose	0	>0.05	Not estimated

CMIN, Chi-square of the model; DF, Degrees of freedom; CMIN/DF, Chi-square ratio to degrees of freedom; CFI, Comparative Fit Index; RMSEA, Root Mean Square Error of Approximation; PClose, Test of model fit closeness.

The results presented in Table 4 allow an evaluation of the adequacy of the confirmatory factor model used to analyze the dimensions associated with the use of clinical simulators in higher education. Structural equation modeling has been widely applied in studies examining the adoption and effectiveness of technological tools in medical education, as it allows researchers to identify relationships between latent constructs and observed indicators within complex learning environments (28). From a methodological perspective, the ratio CMIN/DF = 3.171 indicates an acceptable level of model fit, since values between 1 and 3 are generally considered indicative of a reasonable correspondence between the theoretical model and the empirical data. This interpretation is consistent with methodological studies that emphasize the importance of evaluating multiple fit indices when validating measurement models in educational research (29). Consequently, the obtained values suggest that the proposed factorial structure adequately represents the relationships among the observed variables included in the research instrument, supporting the internal coherence of the constructs associated with simulation-based learning.

Similarly, the Comparative Fit Index (CFI = 0.900) reflects an acceptable incremental fit of the model when compared with a null model, indicating that the proposed structure explains a considerable proportion of the covariance among the observed variables. Previous research has demonstrated that structural equation modeling is particularly useful for understanding the factors that influence the adoption and effectiveness of simulation technologies in medical education, where perceived usefulness, usability, and contextual learning conditions interact to shape student engagement (30). In methodological terms, values close to 0.90 have been considered acceptable in applied educational research involving complex models and multiple latent constructs (31). Therefore, the obtained CFI value supports the theoretical consistency of the relationships between the constructs analyzed in this study and suggests that the model adequately explains the influence of clinical simulation on the development of practical competencies in higher education contexts.

However, the Root Mean Square Error of Approximation (RMSEA = 0.098) indicates a moderate level of approximation error, suggesting that the model fit could be improved through further refinement of the measurement structure. Simulation-based learning environments are inherently multidimensional, integrating cognitive, technical, and experiential components that may influence the statistical behavior of structural models (32). From a methodological standpoint, several studies have indicated that the interpretation of model fit should consider the joint evaluation of multiple indices rather than relying on a single parameter (33, 34). In this sense, although the RMSEA value suggests room for improvement, the overall interpretation of the indices indicates that the model presents an acceptable but improvable fit, supporting the construct validity of the instrument while highlighting the importance of continuous methodological validation in studies that evaluate educational technologies in healthcare training.

Table 5 represents the final structural model obtained through confirmatory factor analysis, integrating latent constructs and the relationships among the theoretical dimensions of the instrument. This model allows for examining the convergent and discriminant validity of variables associated with the use of clinical simulators, as well as the statistical robustness of the estimated relationships among factors.

**Table 5:** Evaluation of the structural model for convergent and discriminant validity of the instrument on the use of clinical simulators.

Dimensions	CR	AVE	MSV	MaxR(H)	D1	D2	D3	D4	D5
D1. Access and use of clinical simulators	0.896	0.594	0.679	0.918	**0.771**				
D2. Acquisition of technical competencies	0.969	0.799	0.782	0.972	0.824^***^	**0.894**			
D3. Teamwork and communication	0.966	0.826	0.790	0.967	0.745^***^	0.884^***^	**0.909**		
D4. Safety and confidence in clinical performance	0.952	0.797	0.790	0.953	0.696^***^	0.823^***^	0.889^***^	**0.893**	
D5. Overall perception and formative impact	0.948	0.784	0.645	0.953	0.528^***^	0.654^***^	0.746^***^	0.803^***^	**0.885**

CR, Composite Reliability; AVE, Average Variance Extracted; MSV, Maximum Shared Variance; MaxR(H), Maximum Composite Reliability. The values on the diagonal represent the square root of the AVE, while the off-diagonal values correspond to correlations among the dimensions. Bold values represent the square root of the Average Variance Extracted (AVE) for each construct (diagonal elements), indicating convergent validity. Off-diagonal values correspond to inter-construct correlations. ***p < 0.001 indicates statistically significant correlations at a high confidence level.

The model results, as detailed in Table 5, demonstrate an adequate fit, supporting the internal coherence between the items and the proposed theoretical dimensions. The values obtained for the fit indices (CFI > 0.90; RMSEA < 0.10) reflect a stable factorial structure consistent with the methodological recommendations of Carter et al. (35), who state that models with indices within these ranges exhibit acceptable reliability and strong construct validity in educational and clinical research. Likewise, the confirmation of convergent and discriminant validity aligns with the findings of Bártolo et al. (36), who demonstrated that appropriate factor correlations and construct differentiation are essential to ensure the precision of psychometric instruments.

Similarly, Gajewski et al. (37) argue that second-order confirmatory analysis allows simultaneous evaluation of factorial validity and consistency in hierarchical models, making it a suitable approach for applied studies in professional training contexts. Along these lines, Marsh et al. (38) emphasizes that the assessment of multidimensional models with multiple constructs enables the identification of more precise latent relationships and contributes to improving predictive validity. Therefore, the results of the present model confirm that the theoretical dimensions access, technical competence, teamwork, safety, and overall perception consistently reflect the components of learning mediated by clinical simulators.

Table 6 presents the results of the internal reliability analysis of the instrument applied to measure students’ perceptions of the use of clinical simulators in professional training. This analysis verifies the consistency of the identified theoretical dimensions, ensuring the precision and stability of the measurements conducted within the educational context.

**Table 6:** Internal reliability and consistency analysis of the dimensions of the instrument on the use of clinical simulators.

Dimensions	D1. Access and use of clinical simulators	D2. Acquisition of technical competencies	D3. Teamwork and communication	D4. Safety and confidence in clinical performance	D5. Overall perception and formative impact
D1. Access and use of clinical simulators					
D2. Acquisition of technical competencies	0.821				
D3. Teamwork and communication	0.748	0.889			
D4. Safety and confidence in clinical performance	0.698	0.826	0.890		
D5. Overall perception and formative impact	0.515	0.659	0.750	0.812	

HTMT, Heterotrait–Monotrait Ratio; D1, Access and use of clinical simulators; D2, Acquisition of technical competencies; D3, Teamwork and communication; D4, Safety and confidence in clinical performance; D5, Overall perception and formative impact. Values below 0.85 indicate adequate discriminant validity among dimensions.

The analysis presented in Table 6 provides evidence of strong internal reliability and adequate discriminant validity across the five dimensions of the instrument assessing the use of clinical simulators. These results are consistent with those obtained by Alconero-Camarero et al. (39), who validated a similar questionnaire on satisfaction in high-fidelity clinical simulations and reported an overall reliability of 0.857, demonstrating that such tools are robust for measuring educational perceptions in simulated environments. Likewise, Sharif-Nia et al. (40) argue that high internal consistency reflects the conceptual coherence of items around a single construct, reinforcing the validity of the confirmatory factor model applied in psychometric studies within health education.

In this regard, the findings of the present research reaffirm that the dimensions “access and use,” “technical competencies,” “teamwork,” “safety,” and “overall perception” exhibit a strong interrelationship that supports a comprehensive evaluation of the impact of clinical simulation. This result aligns with the conclusions of Panzeri et al. (41), who highlighted the relevance of internal consistency as an essential criterion for confirming discriminant validity in structural equation models applied to educational and clinical contexts.

Table 7 presents the proposed structural model representing the hypothesized relationships among the dimensions of the use of clinical simulators and the development of practical skills in obstetrics students. This model enables visualization of the magnitude and direction of relationships among latent constructs, demonstrating the importance of clinical simulation as a pedagogical tool in professional training.

**Table 7:** Structural model of the impact of the use of clinical simulators on the development of practical skills.

Hypothesis	Non-standardized loadings	Standardized loadings (*β*)	*t*	*p*-value	LCI (95%)	UCI (95%)	Threshold/Hypothesis
H1	−0.050	−0.063	−0.86	0.393	−0.166	0.066	Not accepted
H2	−0.038	−0.063	−0.59	0.553	−0.165	0.089	Not accepted
H3	0.245	0.296	2.86	0.005	0.077	0.414	Accepted
H4	0.609	0.613	7.44	0.000	0.448	0.770	Accepted

Standardized coefficients (*β*), *t*-values, and significance levels (*p*) obtained from the validation of the structural model. H1: Access and use of clinical simulators; H2: Acquisition of technical competencies; H3: Teamwork and communication; H4: Safety and confidence in clinical performance. The results indicate that H3 (*β* = 0.296; *p* = 0.005) and H4 (*β* = 0.613; *p* < 0.001) were statistically significant and accepted, while H1 and H2 did not reach significance and were therefore rejected.

The results presented in Table 7 show that the most significant paths correspond to the dimensions “teamwork and communication” (*β* = 0.296; *p* = 0.005) and “safety and confidence in clinical performance” (*β* = 0.613; *p* < 0.001), indicating that these variables exert a direct and positive effect on the overall perception of formative impact. This suggests that clinical simulation not only contributes to the development of technical skills but also strengthens interpersonal and self-confidence competencies essential for professional practice in real-world contexts.

These findings are consistent with those reported by Akalin and Sahin (42), who concluded that obstetric simulation significantly improves nursing students’ self-confidence, critical thinking, and self-efficacy, promoting comprehensive learning oriented toward safe clinical performance. Similarly, Everett et al. (43) emphasizes that simulation in obstetrics and gynecology enhances the transfer of knowledge to practice and increases responsiveness in obstetric emergency situations, thereby strengthening professional development and patient safety.

Moreover, the results support the idea that clinical simulation should be implemented as part of a continuous educational strategy aimed at strengthening both technical and socioemotional competencies. In this regard, Akalin and Sahin (42) and McGaghie et al. (23) agree that simulation-based learning fosters deliberate repetition, formative feedback, and critical reflection key factors for the consolidation of advanced clinical skills.

## 4 Conclusion

The study achieved its objective of analyzing the effects of the use of clinical simulators on the development of practical skills among students of the Professional School of Obstetrics at Universidad Peruana Los Andes, demonstrating the effectiveness of simulation as a formative strategy in higher education within the health sciences. The results showed that the hypotheses related to teamwork and communication, as well as to safety and confidence in clinical performance, were statistically significant, while the hypotheses associated with access, use, and acquisition of technical competencies did not reach significance. This finding confirms that the impact of clinical simulation is mainly reflected in dimensions associated with socioemotional competencies, collaboration, and professional self-confidence.

The research addressed the proposed question by demonstrating that the use of clinical simulators positively influences the overall perception of formative impact, strengthening safety, problem-solving capacity, and performance quality in controlled practice environments. It was demonstrated that simulation contributes to the comprehensive education of students by enabling the application of theoretical knowledge in scenarios that replicate real-life situations, promoting decision-making and reflective practice.

Among the study’s limitations, it is recognized that the research was conducted within a single institutional context and employed a cross-sectional design, which limits the ability to establish long-term causal relationships. Nevertheless, the internal consistency of the instrument and the statistical validation of the structural model support the reliability of the results and the relevance of the adopted approach. Future research is recommended to expand the sample to include different universities and health science programs, as well as to implement longitudinal studies that assess the evolution of clinical competencies throughout the training process. It would also be advisable to incorporate objective performance indicators and triangulation methods that integrate self-assessment with direct observation of simulated practices.

## Data Availability

The datasets presented in this study can be found in online repositories. The names of the repository/repositories and accession number(s) can be found in the article/Supplementary material.

## References

[1] Rooney D Hopwood N Boud D Kelly M. The role of simulation in pedagogies of higher education for the health professions: through a practice-based lens. Vocat Learn. (2015) 8:269–85. doi: 10.1007/s12186-015-9138-z

[2] Weeks KW Coben D O’Neill D Jones A Weeks A Brown M . Developing and integrating nursing competence through authentic technology-enhanced clinical simulation education: pedagogies for reconceptualising the theory-practice gap. Nurse Educ Pract. (2019) 37:29–38. doi: 10.1016/j.nepr.2019.04.010, 31060016

[3] Good ML. Patient simulation for training basic and advanced clinical skills. Med Educ. (2003) 37:14–21. doi: 10.1046/j.1365-2923.37.s1.6.x, 14641634

[4] Cass GKS Crofts JF Draycott TJ. The use of simulation to teach clinical skills in obstetrics. Semin Perinatol. (2011) 35:68–73. doi: 10.1053/j.semperi.2011.01.005, 21440813

[5] Gavin NR Satin AJ. Simulation training in obstetrics. Clin Obstet Gynecol. (2017) 60:802–10. doi: 10.1097/GRF.0000000000000322, 28945614

[6] Costa LA Monger EJ. Criteria to evaluate graduate nurse proficiencies in obtaining a health history and perform physical assessment in simulation-based education: a narrative review. Nurse Educ Pract. (2024) 77:103984. doi: 10.1016/j.nepr.2024.103984, 38678870

[7] Higham H Baxendale B. To err is human: use of simulation to enhance training and patient safety in anaesthesia. Br J Anaesth. (2017) 119:i106–14. doi: 10.1093/bja/aex302, 29161386

[8] Rosen KR. The history of medical simulation. J Crit Care. (2008) 23:157–66. doi: 10.1016/j.jcrc.2007.12.004, 18538206

[9] Pajohideh ZS Mohammadi S Keshmiri F Jahangirimehr A Honarmandpour A. The effects of normal vaginal birth simulation training on the clinical skills of midwifery students: a quasi-experiment study. BMC Med Educ. (2023) 23:353. doi: 10.1186/s12909-023-04319-9, 37208680 PMC10199639

[10] Satin AJ. Simulation in obstetrics. Obstet Gynecol. (2018) 132:199–209. doi: 10.1097/AOG.0000000000002682, 29889745

[11] Cooper S Cant R Porter J Bogossian F McKenna L Brady S . Simulation based learning in midwifery education: a systematic review. Women Birth. (2012) 25:64–78. doi: 10.1016/j.wombi.2011.03.004, 21489894

[12] Puri L Das J Pai M Agrawal P Fitzgerald JE Kelley E . Enhancing quality of medical care in low income and middle income countries through simulation-based initiatives: recommendations of the Simnovate Global Health domain group. BMJ Simul Technol Enhanc Learn. (2017) 3:S15–22. doi: 10.1136/bmjstel-2016-000180

[13] Meza PK Bianco K Herrarte E Daniels K. Changing the landscape of obstetric resident education in low- and middle-income countries using simulation-based training. Int J Gynaecol Obstet. (2021) 154:72–8. doi: 10.1002/ijgo.13526, 33314149

[14] Nieto-Gutierrez W Taype-Rondan A. Self-perceived competence in managing obstetric emergencies among recently graduated physicians from Lima, Peru. BMC Med Educ. (2023) 23:876. doi: 10.1186/s12909-023-04854-5, 37974172 PMC10655440

[15] Cordova E Al-Rousan T Castillo-Angeles M Aftab S Nelson BD. Effect of low-cost interventions on the retention of knowledge and skills following helping babies breathe training. Int J Gynecol Obstet. (2018) 142:248–54. doi: 10.1002/ijgo.12512, 29687893

[16] Shibao Miyasato H Armijo-Rivera S Casas Bueno F Sandoval Barrantes AM Delgado Guevara X Gutiérrez Díaz M . Evaluation of a course for simulation instructors at a Peruvian university. Salud, Ciencia y Tecnología - Serie de Conferencias. (2023) 2:429. doi: 10.56294/sctconf2023429

[17] Hassoulas A Crawford O Hemrom S de Almeida A Coffey MJ Hodgson M . A pilot study investigating the efficacy of technology enhanced case based learning (CBL) in small group teaching. Sci Rep. (2025) 15:15604. doi: 10.1038/s41598-025-99764-5, 40320497 PMC12050317

[18] Alrashidi N Pasay an E Alrashedi MS Alqarni AS Gonzales F Bassuni EM . Effects of simulation in improving the self-confidence of student nurses in clinical practice: a systematic review. BMC Med Educ. (2023) 23:815. doi: 10.1186/s12909-023-04793-1, 37904153 PMC10614341

[19] Coyne E Calleja P Forster E Lin F. A review of virtual-simulation for assessing healthcare students’ clinical competency. Nurse Educ Today. (2021) 96:104623. doi: 10.1016/j.nedt.2020.104623, 33125979

[20] de Oliveira Costa RR Meiros SM Dias Coutinho VR Figueira Veríssimo CM da Moreira Silva MM de Souza Lucena EE. Simulação clínica no desempenho cognitivo, satisfação e autoconfiança na aprendizagem: estudo quase-experimental. Acta Paul Enferm. (2020) 33:1–8. doi: 10.37689/acta-ape/2020AO01236

[21] Vogel K Bernloehr A Willmeroth T Blattgerste J Hellmers C Bauer NH. Augmented reality simulation-based training for midwifery students and its impact on perceived knowledge, confidence and skills for managing critical incidents. Midwifery. (2024) 136:104064. doi: 10.1016/j.midw.2024.104064, 38905862

[22] Nicksa GA Anderson C Fidler R Stewart L. Innovative approach using Interprofessional simulation to educate surgical residents in technical and nontechnical skills in high-risk clinical scenarios. JAMA Surg. (2015) 150:201–7. doi: 10.1001/jamasurg.2014.2235, 25565037

[23] McGaghie WC Issenberg SB Cohen ER Barsuk JH Wayne DB. Does simulation-based medical education with deliberate practice yield better results than traditional clinical education? A Meta-analytic comparative review of the evidence. Acad Med. (2011) 86:706–11. doi: 10.1097/ACM.0b013e318217e119, 21512370 PMC3102783

[24] Coro-Montanet G Pardo Monedero MJ Sánchez Ituarte J Wagner Porto Rocha H Gomar Sancho C. Numerical assessment tool to measure realism in clinical simulation. Int J Environ Res Public Health. (2023) 20:2247. doi: 10.3390/ijerph20032247, 36767618 PMC9916353

[25] Yang F Wang Y Yang C Zhou MH Shu J Fu B . Improving clinical judgment by simulation: a randomized trial and validation of the Lasater clinical judgment rubric in Chinese. BMC Med Educ. (2019) 19:20. doi: 10.1186/s12909-019-1454-9, 30642320 PMC6332860

[26] CooperJB TaquetiVR. A brief history of the development of mannequin simulators for clinical education and training. Postgrad Med J. (2008) 84:563–70. doi: 10.1136/qshc.2004.009886, 19103813

[27] Stunt J Wulms P-B Kerkhoffs G Dankelman J Tuijthof G van Dijk N. How valid are commercially available medical simulators? Adv Med Educ Pract. (2014) 5:385–95. doi: 10.2147/AMEP.S63435, 25342926 PMC4205038

[28] Melas CD Zampetakis LA Dimopoulou A Moustakis V. Modeling the acceptance of clinical information systems among hospital medical staff: an extended TAM model. J Biomed Inform. (2011) 44:553–64. doi: 10.1016/j.jbi.2011.01.009 21292029

[29] Choi H Tak SH. Nurses’ behavioral intention in using virtual clinical simulation training: by structural equation modeling. Nurse Educ Pract. (2022) 65:103492. doi: 10.1016/j.nepr.2022.103492, 36332490

[30] Tang L Ning Y Lv H Li X Tang A. Predictors of medical students’ adoption of emergency medicine virtual simulation platforms. IEEE Access. (2025) 13:19237–49. doi: 10.1109/ACCESS.2025.3531507

[31] Goretzko D Siemund K Sterner P. Evaluating model fit of measurement models in confirmatory factor analysis. Educ Psychol Meas. (2024) 84:123–44. doi: 10.1177/00131644231163813, 38250508 PMC10795573

[32] Mujlli G Al-Ghosen A Alrabah R Munshi F Ozdemir B. Development and validation of simulation scenario quality instrument (SSQI). BMC Med Educ. (2023) 23:972. doi: 10.1186/s12909-023-04935-5, 38115012 PMC10731859

[33] Greve KW Stickle TR Love JM Bianchini KJ Stanford MS. Latent structure of the Wisconsin card sorting test: a confirmatory factor analytic study. Arch Clin Neuropsychol. (2005) 20:355–64. doi: 10.1016/j.acn.2004.09.004, 15797171

[34] Hoofs H van de Schoot R Jansen NWH Kant I. Evaluating model fit in Bayesian confirmatory factor analysis with large samples: simulation study introducing the BRMSEA. Educ Psychol Meas. (2018) 78:537–68. doi: 10.1177/0013164417709314, 30034027 PMC6041765

[35] Carter SR. Using confirmatory factor analysis to manage discriminant validity issues in social pharmacy research. Int J Clin Pharm. (2016) 38:731–7. doi: 10.1007/s11096-016-0302-9, 27147255

[36] Bártolo A Monteiro S Pereira A. Factor structure and construct validity of the generalized anxiety disorder 7-item (GAD-7) among Portuguese college students. Cad Saude Publica. (2017) 33:e00212716. doi: 10.1590/0102-311x00212716, 28977285

[37] Gajewski BJ Boyle DK Miller PA Oberhelman F Dunton N. A multilevel confirmatory factor analysis of the practice environment scale. Nurs Res. (2010) 59:147–53. doi: 10.1097/NNR.0b013e3181d1a71e, 20216017

[38] Marsh HW Morin AJS Parker PD Kaur G. Exploratory structural equation modeling: an integration of the best features of exploratory and confirmatory factor analysis. Annu Rev Clin Psychol. (2014) 10:85–110. doi: 10.1146/annurev-clinpsy-032813-153700, 24313568

[39] Alconero-Camarero AR Romero AG Sarabia-Cobo CM Arce AM. Clinical simulation as a learning tool in undergraduate nursing: validation of a questionnaire. Nurse Educ Today. (2016) 39:128–34. doi: 10.1016/j.nedt.2016.01.027, 27006044

[40] Sharif-Nia H Marôco J Rahmatpour P Ghahrani N Muhammad Ibrahim F Mohammad Ibrahim M . Psychometrics evaluation of the university student engagement inventory in online learning among Arab students. BMC Nurs. (2023) 22:158. doi: 10.1186/s12912-023-01318-5, 37158930 PMC10169459

[41] Panzeri A Castelnuovo G Spoto A. Assessing discriminant validity through structural equation modeling: the case of eating compulsivity. Nutrients. (2024) 16:550. doi: 10.3390/nu16040550, 38398874 PMC10892802

[42] Akalin A Sahin S. Obstetric simulation in undergraduate nursing education: an integrative review. Nurs Forum. (2020) 55:369–79. doi: 10.1111/nuf.12437, 32030752

[43] Everett EN Forstein DA Bliss S Buery-Joyner SD Craig LB Graziano SC . To the point: the expanding role of simulation in obstetrics and gynecology medical student education. Am J Obstet Gynecol. (2019) 220:129–41. doi: 10.1016/j.ajog.2018.10.029, 30696555

[44] World Medical Association. World Medical Association Declaration of Helsinki: ethical principles for medical research involving human subjects. JAMA. (2013) 310:2191–4. doi: 10.1001/jama.2013.281053, 24141714

